# Changes in Electronic Cigarette Use from 2013 to 2015 and Reasons for Use among Finnish Adolescents

**DOI:** 10.3390/ijerph13111114

**Published:** 2016-11-09

**Authors:** Jaana M. Kinnunen, Hanna Ollila, Pirjo L. Lindfors, Arja H. Rimpelä

**Affiliations:** 1 School of Health Sciences, University of Tampere, 33014 Tampere, Finland; pirjo.lindfors@uta.fi (P.L.L.); arja.rimpela@uta.fi (A.H.R.); 2 Department of Health, National Institute for Health and Welfare, 00271 Helsinki, Finland; hanna.ollila@thl.fi; 3 PERLA—Tampere Centre for Childhood, Youth and Family Research, University of Tampere, 33014 Tampere, Finland; 4 Department of Adolescent Psychiatry, Pitkäniemi Hospital, Tampere University Hospital, 33380 Nokia, Finland

**Keywords:** electronic cigarette, electronic nicotine delivery system, adolescents, tobacco use, Finland

## Abstract

Electronic cigarettes are quite a new potential source of nicotine addiction among youth. More research is needed, particularly on e-liquid use and socioeconomic factors as potential determinants. We studied changes from 2013 to 2015 in adolescent e-cigarette awareness and ever-use, types of e-liquids, and determinants in Finland. In 2015, we studied weekly use and reasons for ever-use. Data were from two national surveys of 12–18-year-old Finns (2013, *n* = 3535, response rate 38%; 2015, *n* = 6698, 41%). Descriptive statistics and logistic regression analysis were used. Awareness and ever-use of e-cigarettes increased significantly from 2013 to 2015 in all age and gender groups. Ever-use increased from 17.4% to 25%, with half having tried nicotine e-liquids. In 2015, weekly use was rare (1.5%). Daily cigarette smoking was the strongest determinant (OR 51.75; 95% CI 38.18–70.14) for e-cigarette ever-use, as for e-cigarette weekly use, but smoking experimentation and ever-use of snus (Swedish type moist snuff) and waterpipes alongside parental smoking and poor academic achievement also increased the odds for ever-use. The most common reason behind e-cigarette ever-use was the desire to try something new. To conclude, adolescent e-cigarette ever-use is increasing, and also among never-smokers. Tobacco-related factors are stronger determinants for e-cigarette use than socioeconomic factors.

## 1. Introduction

Awareness and use of electronic cigarettes (e-cigarettes) or electronic nicotine delivery systems (ENDS) has been increasing both among adults and adolescents [1,2,3,4]. E-cigarette experimentation (tried at least once) and more regular use seem to center on younger smokers, but are not limited to them—growing proportions of e-cigarette use among never-smoking adolescents and young adults are being reported [5,6].

Only a handful of studies have specifically inquired about the reasons or motives behind adolescent e-cigarette experimentation or use. Kong et al. [7] found that the top reasons for experimentation among U.S. youth were curiosity, appealing flavors and peer influences. Curiosity has been cited by teenagers as the top reason also in Switzerland [8] and in New Zealand [4]. Top reasons for discontinuation in the U.S. study [7] were losing interest, perceiving e-cigarettes as “uncool” and expressing health concerns. Adolescent cigarette smokers perceived that e-cigarettes can also be used in places where smoking is prohibited, which is what led them to try e-cigarettes. Another reason for adolescent cigarette smokers to try e-cigarettes was to quit smoking. They discontinued their use mostly because e-cigarettes were not as satisfying for them as cigarettes [7].

E-cigarette experimentation and use have been associated among youth with susceptibility to conventional cigarette smoking initiation [6,9], conventional cigarette smoking [4,5,8,10,11,12,13,14,15,16,17], male gender [4,8,12,13,15], ever-use of other tobacco (combustible and non-combustible) [10,11,13], alcohol use [4,8,13,17], perception of low harm of e-cigarettes [18,19,20,21], peer smoking behavior [4,12,17,20], parents’ smoking [20,22], peer e-cigarette use [23] and exposure to e-cigarette advertising [24,25]. Socioeconomic factors have been studied less, and their association with e-cigarette use is not so clear. However, adolescent’s vocational education [8,10], poor academic achievement [10], attending disadvantaged school [15], and parents’ lower educational level [17] have been found to be associated with e-cigarette experimentation and use. Although e-cigarettes are marketed as a smoking cessation tool [26,27], the available cross-sectional studies rarely show any association between e-cigarette use and smoking cessation or intention to quit smoking among youth [4,5,10]. The associated factors with regular use (daily or weekly use) of e-cigarettes, and, as such, have not been studied before (see [8]).

Studies on adolescent e-cigarette use have paid little attention to the actual contents of e-liquids, besides flavors, in studies of adolescent e-cigarette use. Adolescents’ use of e-liquids containing nicotine raises concern, as it may constitute a risk for nicotine dependence, and nicotine may have a lasting effect on adolescents’ developing brains [28,29]. Kinnunen et al. [10] were the first to report that the majority of Finnish adolescent e-cigarette ever-users had indeed used nicotine-containing e-liquids. The e-liquid contents are an important research question not only in terms of preventing nicotine addiction, but also from the perspective of product regulation and youth access laws.

At the time of our surveys in 2013 and 2015, the Finnish tobacco legislation prohibited selling tobacco products to youth under 18 years old, but this did not concern e-cigarettes, as they were classified as tobacco imitations and e-liquids as substitute tobacco. Thus, minors could purchase them freely when they were available in the shops. By contrast, nicotine-containing e-cigarettes and e-liquids were treated as medicinal products, meaning that strict safety and efficacy evidence must have been demonstrated before a selling permit was granted. At the moment of the surveys, no e-cigarette company had a selling permit for nicotine-containing e-liquids in Finland. However, they could be acquired from visits abroad or online by consumers, including adolescents. Most minors indeed reported getting their e-cigarettes mainly from friends or online [10].

In this study, we report changes from 2013 to 2015 in the awareness and use of e-cigarettes and in the use of nicotine and non-nicotine e-liquids using nationally representative data of 12–18-year-old Finns. Furthermore, we investigate whether the determinants for e-cigarette ever-use have changed during the two-year period. We also study regular use of e-cigarettes, determinants for weekly e-cigarette use, and reasons for e-cigarette use in 2015.

## 2. Materials and Methods

### 2.1. Sampling and Participants

This study was based on the nationally representative data collected as part of the 2013 and 2015 Adolescent Health and Lifestyle Survey, which is a cross-sectional postal survey to investigate adolescent health and health behaviors. It has been conducted biennially in Finland since 1977, with an option to answer also via a protected online form since 2009. Nationally representative samples of 12-, 14-, 16- and 18-year-olds were obtained from The Population Register Centre (Helsinki, Finland) [30]. All adolescents born on certain days in June, July or August were selected. The study procedure has been kept similar to enable comparisons between survey years. The Ethics Committee of the Tampere Region, Finland approved the study protocol in 2013 and 2015 (Code: 4/2013 and 31/2014).

Self-administered questionnaires were sent by mail to 9398 adolescents in 2013 and to 16,473 adolescents in February 2015, followed by two reminders to non-respondents. The number of respondents in 2013 was 3535 (response rate 38%), and, in 2015, 6698 (response rate 41%). Girls responded more actively than boys: in 2013, the response rate for girls was 46% and for boys 30%, and, in 2015, 47% and 34%, respectively.

### 2.2. The Measures

The awareness and use of e-cigarettes was assessed in both years by posing the questions: “Have you ever tried electronic cigarettes? How many times altogether?” The options were: “I do not know what they are”, “No”, “I have tried once or twice”, “I have tried 20 times or less” and “I have tried more than 20 times”. For the analysis of determinants for e-cigarette ever-use, answers to options “I do not know what they are” and “No” were classified as never-use of e-cigarettes, and the answers to other options as ever-use. To explore the types of liquids used, the respondents were asked “If you have used electronic cigarettes, what substance did they contain?”, and they could choose one or more of the following options: “Liquid with nicotine”, “Liquid without nicotine” and “I do not know”, and, in 2015, also “Something else, what?” (an open-ended option).

Regular use was estimated only in 2015 with a question: “Which one of the following alternatives best describes your current use of e-cigarettes?” with the options “I do not use e-cigarettes”, “I use e-cigarettes less than once a week”, “I use e-cigarettes once a week or more often, but not daily” and “I use e-cigarettes once a day or more often”. For the analysis of determinants for e-cigarette weekly use, answers to options “I use e-cigarettes once a week or more often, but not daily” and “I use e-cigarettes once a day or more often” were classified as weekly use of e-cigarettes. The question on the reasons for e-cigarette use was: “What were the most important reasons why you tried an e-cigarette or started using them?” The options were “I wanted to try something new”, “I wanted to stop smoking”, “My friends started to use them” and an open-ended “Something else, what?” The respondent could report more than one reason.

In the analysis of determinants, the following tobacco related factors were used: smoking status, snus (Swedish type moist snuff) use, waterpipe use, parents’ smoking, and exposure to e-cigarette advertising, which were all self-reported by the adolescents. The socioeconomic factors were self-reported academic achievement in comparison with class average, family structure, parents’ work situation, and parents’ education, which was categorized according to the highest educational level of the parents.

### 2.3. Analysis of Non-Response

For the analyses of non-response, a shorter questionnaire was sent to the non-respondents. Those who answered the original questionnaire were compared with those who answered the short questionnaire, which was sent with the third reminder to non-respondents. It included the main questions on tobacco and e-cigarette use. It was assumed that the respondents to the short questionnaire represent closely all non-respondents. The number of respondents to the short questionnaire was 623 in 2013 and 714 in 2015. The groups did not differ by e-cigarette ever-use (*p* = 0.502 in 2013 and *p* = 0.393 in 2015), weekly use (*p* = 0.679 in 2015) or by age (*p* = 0.216 in 2013 and *p* = 0.972 in 2015). Boys were more likely to be non-respondents in 2013 (*p* = 0.01), but not in 2015 (*p* = 0.103). Adolescents with low or average academic achievement were more likely to be non-respondents (*p* = 0.024 in 2013 and *p* < 0.001 in 2015).

### 2.4. Data Analysis

Awareness, use and regular use of e-cigarettes were cross-tabulated with age, gender, tobacco use and socioeconomic factors. Direct adjustment, giving equal weights to each group, was used to calculate age- and gender-adjusted prevalence. E-liquids and reasons for e-cigarette use are presented for those who had used e-cigarettes.

Binary and stepwise logistic regression analyses were used to analyze the factors associated with ever-use of e-cigarettes from the pooled data of both years. First, the analysis was conducted separately for all independent variables, including survey year, adjusting for age and gender. Then, all independent variables were included in a multivariate model. The factors associated with weekly use of e-cigarettes were analyzed from 2015 data the same way as for ever-use, but without survey year. The Pearson χ^2^ test was used to test statistical differences. SPSS Statistics V.23 (IBM, Armonk, NY, USA) was used for all data analyses.

## 3. Results

### 3.1. Awareness and Use of E-Cigarettes

Awareness of e-cigarettes increased from 2013 to 2015 in Finland (Table 1). In 2013, 85.3% of the 12–18-year-olds reported knowing what e-cigarettes are, while the proportion was 94.0% in 2015. Overall, 17.4% of the respondents had tried e-cigarettes in 2013, and 25.0% in 2015 (*p* < 0.001). Most adolescents had tried e-cigarettes only once or twice (12.6% in 2013 and 16.4% in 2015). The proportion of those who had tried e-cigarettes more than 20 times had risen from 2.0% to 4.7%. The e-cigarette experimentation and use increased from 2013 to 2015 among both genders. Boys had experimented more often than girls, *p* < 0.001 (Table 1).

Most of those who had tried e-cigarettes had also tried conventional cigarettes (91.5% in 2013 and 83.3% in 2015) (not shown in a table). In 2013, 42.3% of those who had tried conventional cigarettes had also tried e-cigarettes, while the proportion was 64.1% in 2015. Of those who had never tried conventional cigarettes, 2.6% had tried e-cigarettes at least once in 2013 and 6.3% in 2015. Among 12-year-old never-smokers, experimenting was less frequent (0.4% in 2013 and 1.5% in 2015) than among older age groups. In 2015, 0.7% of never-smokers had tried e-cigarettes more than twice, while the proportion was 0% in 2013.

In 2015, 1.5% of all respondents reported e-cigarette use at least weekly, and 3.4% less than once a week. Regular use of e-cigarettes was very rare among 12-year-olds, but it became more common with age (Table 1). Daily use of e-cigarettes was most common among 18-year-old boys (3.9%). The difference in regular use of e-cigarettes between boys and girls was statistically significant (*p* < 0.001).

### 3.2. Nicotine and Non-Nicotine Containing E-Liquids

Among the e-cigarette users, e-liquid containing nicotine was used more often than liquid without nicotine, but its proportion decreased from 2013 to 2015 (Table 2). The proportion of those who did not know the content of the liquid almost doubled from 2013 to 2015. Among those e-cigarette experimenters who had never tried conventional cigarettes, liquids without nicotine were used most often, but one fifth of them had used liquids with nicotine in 2015 (Table 2).

Among all respondents who had never tried conventional cigarettes, 1.3% had used nicotine-containing e-cigarette in 2015, and 0.6% in 2013 (not shown in a table). Among the respondents who were ever-smokers, the corresponding proportions were 35.7% in 2015 and 29.0% in 2013.

In 2015, of those who had tried e-cigarettes once or twice, 38.2% had used liquids with nicotine and 34.2% had used only liquids without nicotine, while, in 2013, the proportions were 59.0% and 26.5%, respectively (not shown in a table). Most of those who had used e-cigarettes more than 20 times had used liquids with nicotine (more than 80% in both years). The highest proportion (27.6% in 2015 and 14.5% in 2013) of those who did not know what e-liquid they had used was among those who had tried e-cigarettes only once or twice.

### 3.3. Reasons for E-Cigarette Experimentation and Use

**Figure 1:**
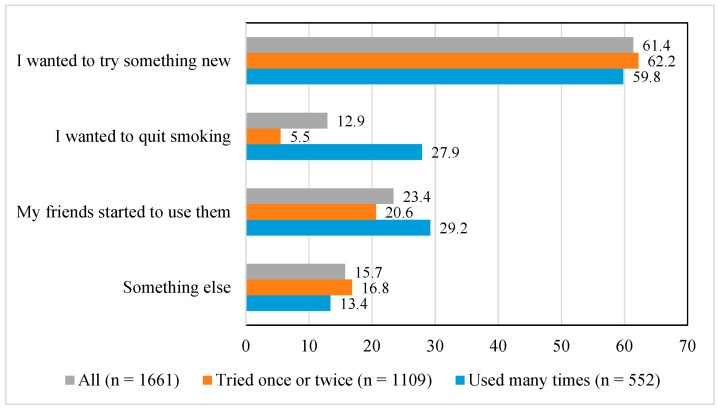
Reasons for e-cigarette experimentation and use among all e-cigarette ever-users and by the frequency of e-cigarette use in 2015, %.

Reasons for e-cigarette experimentation and use were asked in 2015. Adolescents’ most reported reason was “I wanted to try something new”; this was reported by 61.4% of those who had tried e-cigarettes (Figure 1). “My friends started to use them” was reported by 23.4% of e-cigarette experimenters and users, and “I wanted to quit smoking” by 12.9%. Some other reason was given by 15.7%, and it included reasons like “the flavor possibilities”, “an opportunity to try came my way” and “the tricks with smoke/steam”. It was possible to report more than one reason.

Figure 1 shows also the proportions of reasons for e-cigarette experimentation and use among those who had tried e-cigarettes only once or twice and among those who had used them more than that. Those adolescents who had used e-cigarettes more than just a couple of times reported more often that “I wanted to quit smoking” and “My friends started to use them” than those who had experimented only once or twice.

### 3.4. Determinants for E-Cigarette Use

Table 3 presents the determinants for e-cigarette ever-use. In Model 1 (adjusted for age, gender and survey year), along with male gender, all tobacco-related and socioeconomic background variables, excluding father’s work situation, had a significant association with e-cigarette experimentation in pooled data of both survey years (Table 3). The strongest associations were observed for daily smoking and smoking experimentation, followed by snus and waterpipe use. Exposure to e-cigarette advertisements and parents’ smoking were also positively associated with e-cigarette experimentation. Among socioeconomic characteristics, adolescents’ academic achievement was more strongly related to e-cigarette use than family structure, parents’ education or parents’ working situations.

In the final model (Model 2, Table 3) adjusting for all variables, e-cigarette use showed the strongest association with conventional cigarette smoking, followed by snus and waterpipe use, and male gender. Among socioeconomic characteristics, only having poorer academic achievement retained a positive significant association with e-cigarette use. The interactions between each variable in Model 2 and the survey year were also tested. The only interaction that remained statistically significant in the final Model 2 was the interaction between waterpipe use and the survey year. The odds ratios for e-cigarette use among those who had tried waterpipe were 6.54 (95% CI 5.27–8.12) in 2013, and 9.66 (8.15–11.45) in 2015.

Table 4 presents the determinants for weekly use of e-cigarettes in 2015. In Model 1 in Table 4 (adjusted for age and gender), daily smoking, smoking experimentation, snus and waterpipe use, parents’ smoking, academic achievement, family structure and male gender had a significant association with e-cigarette weekly use (Table 4). In Model 2, daily smoking, smoking experimentation, snus use, male gender and father’s smoking only retained significant positive associations with e-cigarette weekly use.

## 4. Discussion

Awareness and use of e-cigarettes increased from 2013 to 2015 among Finnish adolescents. In 2015, only 6% did not know what e-cigarettes were. A quarter of adolescents (25%) had tried e-cigarettes in 2015, compared to 17% in 2013, but weekly use was still rare. Half of e-cigarette users had used e-liquids with nicotine, but one fifth did not know what e-liquid they had used. In addition, some adolescents who had never tried conventional cigarettes had tried nicotine-containing e-cigarettes (1.3% of all never-smokers). The proportion among all ever-smokers was 36.9%. The most common reason to try e-cigarettes was the desire to try something new; only one fourth of those who had used e-cigarettes more than twice reported quitting smoking as the reason to try e-cigarettes. Adolescent e-cigarette ever-use was associated with conventional cigarette smoking, and snus and waterpipe use, but also with lower socioeconomic background, most of all with academic achievement. Weekly use of e-cigarettes was associated significantly only with tobacco-related factors.

The results are in line with other studies concerning adolescent e-cigarette use. The proportion of e-cigarette experimenters in 2014 was also one fourth in Sweden, a neighboring country of Finland, and half of the adolescents had used liquids containing nicotine [31]. A recent study from U.S. [32] reported 20% of 12th and 10th graders, and 13% of 8th graders using vaporizer including nicotine at last use, which is less than in our study. Increase in e-cigarette use has been reported also from Poland [33], New Zealand [4] and the United States [34]. Regular use (at least monthly) of e-cigarettes among adolescents was rare also in a study from the UK (2%) [35]. The determinants for e-cigarette use in our study are in line with those reported in other studies—for example conventional cigarette smoking [36] and other tobacco product use [11,13]. In this study, socioeconomic factors were associated with e-cigarette experimentation when studied separately, but when including them in the same model with tobacco-related factors, only adolescent academic achievement remained as a statistically significant determinant. Our study did not confirm parents’ lower educational level as a significant determinant for e-cigarette use that was found in Kaleta et al. [17]. Curiosity has been detected as an adolescents’ most common reason for e-cigarette experimentation in previous studies [4,7,8], which has a similar meaning as a desire to try something new. Friends’ influences have also been reported previously [7]. Our new results on determinants for weekly e-cigarette use revealed that the determinants were the same as for e-cigarette ever-use, and that only tobacco-related factors with male gender were significantly associated with it when included in the same model with socioeconomic background factors. However, weekly e-cigarette use was rare (*n* = 94) leading to large confidence intervals, so these results have to be interpreted with caution, and more research is needed.

Along with e-cigarettes, there are also other new ENDS products, like e-hookahs or e-shishas and vape pens. There is no clear classification system between them, and youth seem to use product characteristics like nicotine content and chargeability when they try to classify these different ENDS products [37]. The contents and types of products also seem to have an impact on the perceived appeal of the product or the user prototype. For instance, e-hookah users are perceived as young and trendy but e-cigarette users as old and addicted to nicotine [37].

The new ENDS products may well be replacing conventional cigarettes in adolescents’ smoking experimentations. In the 2015 Adolescent Health and Lifestyle Survey [38], the age and gender adjusted prevalence of tried conventional cigarette smoking among 12–18-year-olds was 32%, only seven percentage units higher than the prevalence of tried e-cigarettes (25%). It remains to be seen whether e-cigarettes are here to stay, and adolescents increasingly experiment with them and decreasingly with conventional cigarettes, or whether e-cigarettes are just a passing fad for youth.

In Finland, minors have been able to buy non-nicotine e-cigarettes from shops without any age limits, and nicotine e-liquids from the internet. Usually, adolescents have obtained these products from friends [10]. As the Finnish Tobacco Act has been revised in 2016 to comply with the new European Union Tobacco Products Directive (2014/40/EU) and also to introduce new national regulations [39], this situation is now about to change. According to the new legislation, e-cigarettes will be subject to the same regulations as tobacco products, including sales prohibition to minors (18 years), point-of sale display ban and non-vaporing areas [39]. Adolescent smoking has decreased in Finland over a decade [38], but the nicotine dependence may not diminish if e-cigarettes will substitute adolescents’ use of conventional cigarettes.

A few qualitative studies suggest that adolescents themselves seem to support strong e-cigarette regulations and endorse restrictions on sales to minors, marketing and e-cigarette use in public places. In their study, Weishaar et al. [40] found that concern about potential health harms of e-cigarette use and marketing increasing the acceptability of vaping and smoking led adolescents to support regulation. Adolescents seem to be well aware of the current debates around e-cigarettes. Participants critically considered existing evidence and competing interests in regulatory debates and demonstrated a sophisticated understanding of the advantages and disadvantages of regulations. Another qualitative study also showed that youth were able to point out several aspects on how the products could be used in smoking cessation, but did not generally perceive that e-cigarette use leads to successful quitting experiences [41].

This study has some limitations. The low response rates may compromise the generalizability of the study, but the indirect comparisons of the respondents and non-respondents found no meaningful differences in the use of e-cigarettes. However, adolescents with higher academic achievement participated more actively, which may have lowered slightly the prevalence of e-cigarette use. The response rates between the years 2013 and 2015 were very similar. If there is a bias, it is similar in both years and the comparison between the years is still valid. Some of the key questions used in the questionnaire may have been limited in scope and may not have captured all of the possible responses, e.g., the question concerning the reasons for e-cigarette use. However, the results on reasons were very similar compared to other studies, so the question has been adequate enough. The question was also piloted before the survey. Additionally, we were not able to investigate all possible determinants for e-cigarette use, and the number of e-cigarette weekly users was small, weakening the generalizability of the results. The validity of self-report of e-cigarette use can be compared to the validity of self-report of conventional cigarette smoking, which has been reported to be good [42]. The strength of our study is that the survey design, instruments, time of data gathering and age groups have been kept the same, enabling comparison between the years. In addition, the number of respondents is large.

## 5. Conclusions

Adolescents’ awareness of e-cigarettes is broad in Finland, and use of and experiments with e-cigarettes are increasing, but weekly use is still rare. Mostly, adolescents use e-cigarettes together with other tobacco products, not to quit smoking. The most common reason behind e-cigarette ever-use was the desire to try something new. This, alongside the associations with ever-use of non-cigarette tobacco, in this study snus and waterpipe, indicates that the use of the products is closely tied to novelty-seeking behavior among youth. Tobacco-related factors are stronger determinants for e-cigarette use than socioeconomic factors. Nicotine-containing e-cigarettes are also quite commonly used and tried by those who have never tried conventional cigarettes. This shows that e-cigarettes can pose a risk for nicotine addiction, not only for smokers, but even for those without a previous history with conventional tobacco products. The use of e-cigarettes, and particularly use of nicotine liquid in them, should be included in all monitoring systems of adolescent health behavior all over the world.

## Figures and Tables

**Table 1:** Distribution of e-cigarette experimentation among adolescents in Finland in 2013 and 2015 and *p*-value for differences between years, and regular use in 2015, by gender and age, %. The total columns are adjusted for age.

**Boys|Age**	**12**		**14**		**16**		**18**		**Total, %**	
**Year**	**2013**	**2015**	**2013**	**2015**	**2013**	**2015**	**2013**	**2015**	**2013**	**2015**
***Ever-use of e-cigarettes***										
Do not know what they are	29.3	13.9	9.9	3.7	4.9	1.6	5.8	1.3	12.5	5.1
Never tried	68.4	81.8	70.6	74.1	66.6	57.8	62.8	53.3	67.1	66.8
Have tried once or twice	1.6	3.7	15.3	15.9	19.5	24.6	20.6	24.3	14.3	17.1
Have tried 20 times or less	0.8	0.3	3.2	3.4	3.7	7.0	5.1	7.3	3.2	4.5
Have tried more than 20 times	-	0.3	1.0	2.9	5.3	9.1	5.8	13.8	3.0	6.5
**Total, %**	100	100	100	100	100	100	100	100	-	-
***n***	256	649	405	893	431	703	277	593	1369	2838
*p*-value	<0.001		<0.001		<0.001		<0.001		<0.001	
Regular use of e-cigarettes *										
I do not use e-cigarettes regularly		99.5		96.3		89.6		89.8		93.8
Less than once a week		0.5		2.6		7.1		5.4		3.9
Once a week or more often but not daily		-		0.6		1.9		0.9		0.9
Once a day or more often		-		0.6		1.3		3.9		1.5
**Total, %**		100		100		100		100		-
***n***		612		854		673		571		2710
**Girls|Age**	**12**		**14**		**16**		**18**		**Total, %**	
**Year**	**2013**	**2015**	**2013**	**2015**	**2013**	**2015**	**2013**	**2015**	**2013**	**2015**
***Ever-use of e-cigarettes***										
Do not know what	43.8	20.6	13.3	3.8	7.2	2.0	3.2	0.7	16.9	6.8
they are										
Never tried	55.9	77.9	74.5	82.6	72.7	66.6	73.2	59.0	69.1	71.5
Have tried once or twice	0.3	1.5	9.1	9.0	15.3	21.4	18.4	30.3	10.8	15.6
Have tried 20 times or less	-	-	2.5	2.3	3.2	5.4	3.7	5.4	2.4	3.3
Have tried more than 20 times	-	-	0.7	2.4	1.7	4.7	1.6	4.6	1.0	2.9
**Total, %**	100	100	100	100	100	100	100	100	-	-
***n***	288	678	596	1090	596	1075	626	952	2106	3795
*p*-value	<0.001		<0.001		<0.001		<0.001		<0.001	
Regular use of e-cigarettes *										
I do not use e-cigarettes regularly		99.7		96.8		94.0		94.4		96.2
Less than once a week		0.3		2.4		4.9		4.0		2.9
Once a week or more often but not daily		-		0.8		1.0		1.1		0.7
Once a day or more often		-		0.1		0.1		0.5		0.2
**Total, %**		100		100		100		100		-
***n***		649		1053		1052		929		3683

* Not asked in 2013.

**Table 2:** Distribution of e-cigarette liquids among all e-cigarette users and among those e-cigarette users who had tried and those who had never tried conventional cigarettes in 2013 and 2015, %.

	Tried Conventional Cigarettes, %		Never Tried Conventional Cigarettes, %		All E-Cigarette Users, %	
Type of Liquid|Year	2013	2015	2013	2015	2013	2015
Liquids with nicotine	69.3	55.9	22.2	21.1	65.3	50.2
Only liquids without nicotine	20.4	25.2	59.3	52.4	23.5	29.7
Do not know	10.4	18.8	18.5	26.5	11.1	20.0
**Total, %**	100	100	100	100	100	100
***n***	579	1375	54	275	637	1661
*p*-value	<0.001		0.454		<0.001	

**Table 3:** Age and gender adjusted prevalence (%) of e-cigarette ever-use and odds ratios (OR) and the 95% confidence interval for e-cigarette use by gender, and tobacco related and socioeconomic factors, among 14–18-year-olds in the pooled data of 2013 and 2015.

Characteristics	*n*	Ever-Use of E-Cigarettes, %	Model 1 *	Model 2 ^†^
			OR (95% CI)	OR (95% CI)
**Survey year**				
2013	2931	22.6	1.00	1.00
2015	5306	32.3	**1.67** (1.50–1.86)	**2.42** (2.00–2.92)
**Age**				
14	2984	17.3	1.00	1.00
16	2805	31.8	**2.27** (2.00–2.58)	1.09 (0.91–1.31)
18	2448	37.3	**2.95** (2.60–3.36)	**0.74** (0.61–0.91)
**Gender**				
Girl	4935	24.8	1.00	1.00
Boy	3302	32.8	**1.52** (1.38–1.68)	**1.48** (1.27–1.72)
**Tobacco related factors**				
**Smoking status**				
Never	4719	7.2	1.00	1.00
Experimenter	2578	48.2	**14.93** (12.85–17.34)	**8.39** (7.03–10.00)
Daily smoker	848	87.5	**126.15** (98.58–161.43)	**51.75** (38.18–70.14)
**Snus use**				
Never	6547	16.2	1.00	1.00
Ever	1676	72.5	**13.14** (11.50–15.01)	**3.14** (2.64–3.75)
**Waterpipe use**				
Never	6701	19.9	1.00	1.00
Ever	1489	67.9	**8.28** (7.25–9.45)	**2.10** (1.57–2.80)
**Parents’ smoking**				
Neither of them smokes	5703	24.8	1.00	1.00
Only mother smokes	552	38.8	**2.07** (1.72–2.50)	1.00 (0.75–1.32)
Only father smokes	1070	35.4	**1.80** (1.56–2.09)	**1.28** (1.04–1.57)
Both of them smoke	564	43.5	**2.67** (2.22–3.21)	**1.40** (1.06–1.85)
**Has seen e-cigarette advertisement**				
No	7368	28.2	1.00	n. s.
Yes	729	38.2	**1.72** (1.46–2.03)	
**Socioeconomic background**				
**Academic achievement**				
Much or slightly better	4050	22.6	1.00	1.00
About class average	3178	31.9	**1.68** (1.51–1.88)	**1.24** (1.06–1.45)
Slightly or much poorer	876	46.2	**3.30** (2.82–3.87)	**1.60** (1.26–2.03)
**Family structure**				
Intact family	6430	26.9	1.00	n. s.
Other family type	1752	35.4	**1.56** (1.38–1.75)	
**Parents’ educational level**				
High	2956	25.4	1.00	n. s.
Middle	4649	30.6	**1.32** (1.18–1.47)	
Low	136	31.4	**1.50** (1.02–2.20)	
**Father’s work situation**				
Working	6856	28.7	1.00	n. s.
Not working	1007	29.0	1.02 (0.87–1.18)	
**Mother’s work situation**				
Working	7161	28.4	1.00	n. s.
Not working	873	32.6	**1.30** (1.12–1.52)	

* Model 1: Logistic regression, adjusted for age, gender and survey year; ^†^ Model 2: Stepwise forward logistic regression; includes all variables in Model 1. Note. Odds ratio (OR) is given in boldface when it indicates a statistically significant (*p* < 0.05) difference from the odds of the reference category. n. s. = not significant.

**Table 4:** Age and gender adjusted prevalence (%) of e-cigarette weekly use and odds ratios (OR) and the 95% confidence interval for e-cigarette use by gender and tobacco related and socioeconomic factors, among 14–18-year-olds in 2015.

Characteristics	*n*	Weekly Use of E-Cigarettes, %	Model 1 *	Model 2 ^†^
			OR (95% CI)	OR (95% CI)
**Survey year**				
2015	5132	2.1		
**Age**				
14	1907	1.1	1.00	n. s.
16	1725	2.2	**2.05** (1.16–3.63)	
18	1500	3.2	**3.07** (1.77–5.31)	
**Gender**				
Girl	3034	1.2	1.00	1.00
Boy	2098	3.1	**2.62** (1.71–4.00)	**2.58** (1.54–4.31)
**Tobacco related factors**				
**Smoking status**				
Never	3018	0.4	1.00	1.00
Experimenter	1553	2.7	**9.87** (4.52–21.52)	**5.38** (2.07–13.98)
Daily smoker	495	13.8	**43.36** (19.65–95.71)	**17.81** (6.48–48.97)
**Snus use**				
Never	4053	0.8	1.00	1.00
Ever	1075	6.0	**7.25** (4.52–11.64)	**2.98** (1.58–5.64)
**Waterpipe use**				
Never	4153	1.2	1.00	n. s.
Ever	955	5.7	**5.00** (3.20–7.82)	
**Parents’ smoking**				
Neither of them smokes	3564	1.4	1.00	1.00
Only mother smokes	348	3.1	**2.28** (1.13–4.60)	1.27 (0.56–2.86)
Only father smokes	656	4.4	**2.94** (1.74–4.96)	**2.14** (1.18–3.87)
Both of them smoke	319	6.1	**4.38** (2.43–7.90)	2.02 (1.00–4.09)
**Has seen e-cigarette advertisement**				
No	4697	2.1	1.00	n. s.
Yes	385	2.7	1.49 (0.77–2.91)	
**Socioeconomic background**				
**Academic achievement**				
Much or slightly better	2546	1.2	1.00	n. s.
About class average	1994	2.6	**2.21** (1.35–3.62)	
Slightly or much poorer	550	4.4	**3.84** (2.14–6.91)	
**Family structure**				
Intact family	4017	1.7	1.00	n. s.
Other family type	1080	3.5	**2.14** (1.39–3.30)	
**Parents’ educational level**				
High	1887	1.5	1.00	n. s.
Middle	2885	2.3	1.51 (0.94–2.44)	
Low	70	1.7	1.08 (0.14–8.15)	
**Father’s work situation**				
Working	4311	2.1	1.00	n. s.
Not working	626	1.9	0.95 (0.50–1.80)	
**Mother’s work situation**				
Working	4478	2.1	1.00	n. s.
Not working	550	2.8	1.34 (0.74–2.43)	

* Model 1: Logistic regression, adjusted for age and gender; ^†^ Model 2: Stepwise forward logistic regression; includes all variables in Model 1. Note: Odds ratio (OR) is given in boldface when it indicates a statistically significant (*p* < 0.05) difference from the odds of the reference category. n. s. = not significant.
